# A Comparative Study of Acute Gastroenteritis Symptoms in Single- versus Multiple-Virus Infections

**DOI:** 10.3390/ijms24098364

**Published:** 2023-05-06

**Authors:** Toshiyuki Hikita, Tung Phan, Shoko Okitsu, Satoshi Hayakawa, Hiroshi Ushijima

**Affiliations:** 1 Hikita Pediatric Clinic, Kiryu City 376-0035, Gunma, Japan; 2 Department of Pathology, University of Pittsburgh, Pittsburgh, PA 15260, USA; 3 Division of Microbiology, Department of Pathology and Microbiology, Nihon University School of Medicine, Bunkyo City 113-8602, Tokyo, Japan

**Keywords:** single-virus infection, multiple-virus infection, acute gastroenteritis, children, Japan

## Abstract

Many different enteric viruses can cause acute gastroenteritis in humans worldwide. While a single virus can indeed cause disease, multiple-virus infections are commonly reported. However, data regarding a comparison between single- and multiple-virus infections upon clinical manifestations of acute gastroenteritis are relatively limited. In this study, a total of 2383 fecal specimens were collected from children with acute gastroenteritis during June 2014–July 2017 in a pediatric clinic in Japan and tested for 11 viruses by multiplex RT-PCR. At least 1 virus was found in 1706 (71.6%) specimens and norovirus GII was the most frequent agent, followed by rotavirus A and other viruses. Multiple-virus infections were identified in 565 cases (33.1%). While major clinical symptoms were found to be significantly different in some single- vs. multiple-virus infections, the disease severity was statistically non-significant. Our study highlights the burden of multiple-virus infections for acute gastroenteritis and the clinical features of patients with multiple-virus infections.

## 1. Introduction

Acute gastroenteritis (AGE) is a major cause of morbidity and mortality, particularly in children and the elderly. While mortality rates declined quite rapidly in many countries, AGE is still responsible for an estimated 533,768 deaths in children under 5 years old [1]. AGE is a common disease, causing a combination of fever, nausea, vomiting, diarrhea, and abdominal pain [2]. Many viruses have been shown to cause acute gastroenteritis; however, studies on the viral etiology of AGE in children have focused mainly on rotavirus A (RVA) and norovirus genogroup I and II (NoV GI and GII) [3,4,5,6]. RVA has a predilection for young children, and RVA infections tend to peak during winter [6]. Globally, approximately 258 million AGE cases in children under 5 years are attributable to RVA infection [7]. NoV affects people of all ages, and it is the common cause of community-acquired diarrhea and AGE outbreaks worldwide [5]. In the United States, NoV has caused approximately 60% of AGE outbreaks. NoV outbreaks often occur during the winter season and in congregate settings [5]. Human adenovirus (HAdV), human astrovirus (HastV), enterovirus (EV), rotavirus B (RVB), rotavirus C (RVC), and sapovirus (SaV) are generally less common compared to RVA or NoV [3,4,5,6]. Enteric infections with parechovirus A (PeV-A) and Aichi virus (AiV) have been reported; however, their reported prevalence varies from place to place [8,9,10]. Multiple-virus infections have been commonly reported in many previous studies [11,12,13,14]. In a molecular epidemiological study on 1010 hospitalized children with AGE in Vietnam, RVA was the most common, with a proportion of 67.4%, and NoV GII was also found in 5.5%. Out of 1010 (5.5%) fecal specimens, 56 were found to be positive with more than one viral agent [15]. Chen et al. (2013) reported that RVA (25.5%) and NoV (18.1%) are the leading etiologies causing AGE in 811 outpatient children in China [16]. In addition, multiple-virus infections were common, and RVA played a major role in these multiple-virus infections [16]. Another molecular epidemiological study on 312 children hospitalized for AGE in Northern India showed that RVA was the most predominant pathogen (18.3%), followed by HAstV (12.5%) and HAdV (9.9%). It was found that multiple-virus infections were detected in 10.6% [17]. Japhet and colleagues (2019) reported that at least one enteric virus was identified in 58.3% of 103 diarrheal fecal specimens in Nigeria [18]. RVA and NoV were detected in 39.8% and 10.7%, respectively. Multiple-virus infections with RVA were observed either with NoV, HAstV, or AiV [18]. At least one viral agent was detected in 48.6% of fecal specimens over an 11-year surveillance in Italy [12]. RVA was the most predominant (24.7%), followed by NoV (19.6%), HAdV (5.3%), and HAstV (3.0%). Multiple-virus infections were discovered in 8.3% of the positive specimens, with common combination being RVA with NoV or with HAstV [12]. However, clinical manifestations in such studies have not been well described because of the difficulty of categorizing symptom data collected from different clinical centers in a consistent manner. In the present study, we performed a comparative study of AGE symptoms in children with single- versus multiple-virus infections in Japan.

## 2. Results

**Figure 1:**
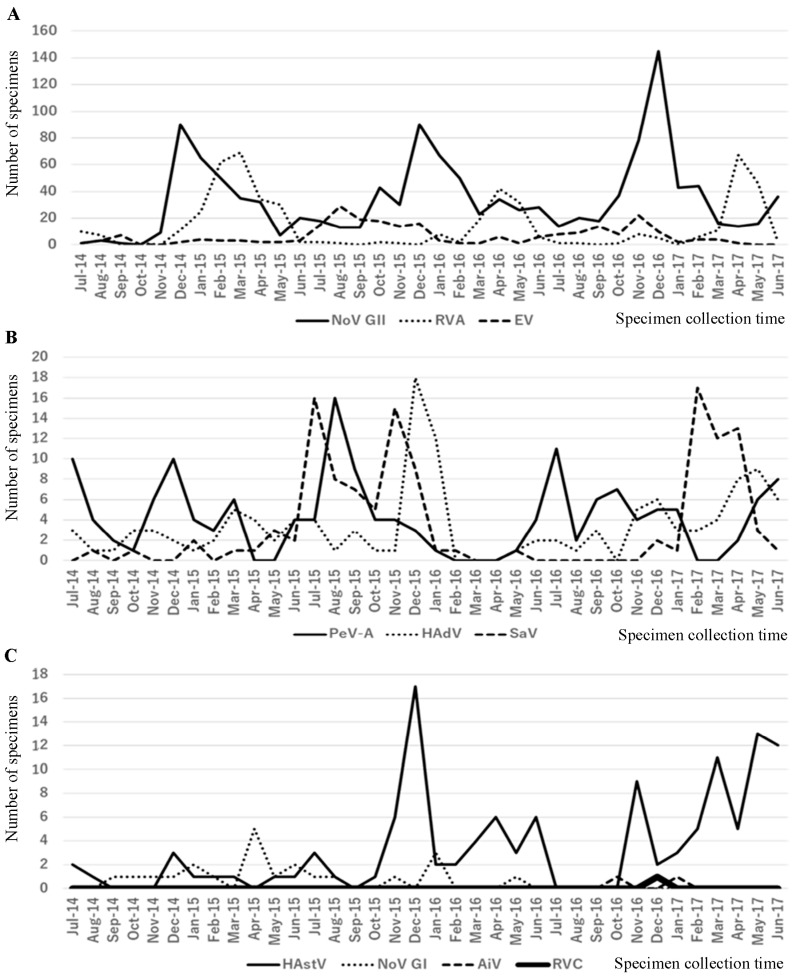
Month-by-month detection frequencies of ten viruses (**A**). NoV GII, RVA, and EV, (**B**) PeV-A, HAdV, and SaV, (**C**) HAstV, NoV GI, AiV, and RVC detected in feces from children with diarrhea who visited Hikita Pediatric Clinic in Japan from June 2014 to July 2017.

A total of 2383 fecal specimens were collected from 1245 children with AGE during July 2014–June 2017 and tested for enteric viruses. It was found that 1706 were positive for 1 or more viruses. NoV GII, the most common virus, was detected in 1229 fecal specimens, followed by RVA and EV in 514 and 241 fecal specimens, respectively (Table 1). Other viruses detected, in a descending order, were PeV-A (152), HAdV (124), SaV (123), HAstV (122), NoV GI (22), AiV (2), and RVC (1). RVB was not found. In each type of virus detected, multiple-virus infections were more common than single-virus infections; however, only NoV GII had a higher percentage of single-virus infections. RVC or AiV was detected in only one or two cases, respectively, and they were co-infected with other viruses (Table 1). Month-by-month detection frequencies of the above ten viruses are shown in Figure 1. NoV GII was detected during all seasons, but it showed peaks in December of each year. RVA was mostly detected in March–April of each year, and at lower frequencies in other months. Other viruses exhibited no clear annual seasonality.

Pairwise comparisons of major symptoms (fever, abdominal pain, vomiting, or diarrhea) were performed for some single-virus infections and multiple-virus infections, which were the most commonly detected, as shown in Table 2. The significant differences in fever (*p* = 0.008), abdominal pain (*p* = 0.036), and diarrhea (*p* = 0.001) were observed between NoV GII and RVA; however, there was no significant difference in vomiting (*p* = 0.1) between NoV GII and RVA. It is interesting that the significant differences in vomiting were seen between NoV GII and other viruses, including EV (*p* = 0.0038), PeV-A (*p* < 0.001), and HAdV (*p* = 0.015). Similar to NoV GII vs. RVA (*p* = 0.001), significant differences in diarrhea were also seen between NoV GII vs. PeV-A (*p* = 0.0001) and NoV GII vs. HAdV (*p* = 0.0009), except NoV GII vs. EV (*p* = 0.73). Pairwise comparisons of the major symptoms’ characteristics were also performed for common single-virus infections and multiple-virus infections, as shown in Table 3. The differences in both the maximum (*p* < 0.0001) and duration (*p* = 0.0024) of fever were significant in only EV vs. NoV GII. There were significant differences in both the frequency and duration of vomiting in four groups, including EV vs. NoV GII, EV/NoV GII vs. NoV GII, PeV-A/NoV GII vs. PeV-A, and PeV-A vs. NoV GII. In addition, both the frequency and duration of diarrhea were significantly different in three groups, including RVA/NoV GII vs. NoV GII, RVA vs. NoV GII, and PeV-A vs. NoV GII. Disease severity was assessed using a clinical Vesikari score, and its median score was four or five for these common single-virus infections and multiple-virus infections. Disease severity was considered to be statistically non-significant (Table 3).

## 3. Discussion

Lu et al. (2015) performed an epidemiological study in outpatient children with AGE in China and reported that RVA (43.3%) was the most prevalent virus, followed by NoV GII (28.9%) [19]. In another study in Korea, the prevalence of RVA infection (17.1%) was higher than that of NoV GII (5.0%) [20]. Interestingly, a high detection rate of NoV GII infection (51.6%) was observed in our study, and RVA was found at a lower detection rate (21.6%). Two major vaccines, Rotarix^®^ (a monovalent G1P [7] live-attenuated human rotavirus vaccine, GlaxoSmithKline Biologicals) and RotaTeq^®^ (a pentavalent G1, G2, G3, G4, P [7] live-attenuated human-bovine mono-reassortant vaccine, Merck & Co., Inc., Rahway, NJ, USA), are licensed and in routine use in Japan. Rotavirus infections have decreased because these rotavirus vaccines have been introduced into national immunization. Therefore, NoV GII became the most common virus in our study. Regarding single and multiple detections, 66.9% of 1706 positive specimens contained 1 virus and 33.1% contained 2 or more viruses. It was found that multiple detections were common between NoV GII and other viruses, notably RVA (34.7%). Of note, a maximum of four viruses were simultaneously detected in three specimens. Similar observation was also seen in an epidemiological study of enteric viruses in Burkina Faso, where multiple-virus infections, including double, triple, and quadruple detections, accounted for 35.7% [21]. Taken together, the data confirm the utility of multiplex RT-PCR assays to detect simultaneously many viruses associated with AGE. With an increasing capacity to efficiently analyze fecal specimens for the presence of different viruses using molecular approaches, there is more evidence of multiple-virus infections in patients.

While there are many studies reporting multiple-virus infections in feces collected from patients presenting with AGE worldwide, the clinical relevance of multiple-virus infections compared to that of single-virus infections is very limited. In our study, significant differences were found for only certain single- and multiple-virus infection comparisons. Abdominal pain and diarrhea were significantly different in HAdV/NoV GII vs. HAdV (*p* = 0.047 and *p* = 0.0088, respectively); fever and vomiting were not in this combination (*p* = 0.64 and *p* = 0.25, respectively), as shown in Table 2. While RVA/NoV GII vs. NoV GII showed significant differences in both the frequency and duration of diarrhea (*p* = 0.0004 and *p* = 0.017, respectively), other symptoms (fever and vomiting) were statistically non-significant (Table 3). It is obvious that the relationship between clinical symptoms and single- vs. multiple-virus infections differed by enteric viruses. In single-virus infections, the clinical symptoms of AGE are associated with a single virus; however, the outcome of infection can be influenced by the combined contribution of multiple viruses in multiple-virus infections. Despite the fact that multiple-virus infections in human feces have been increasingly detected, little is known about their impact on disease outcomes. In multiple-virus infections, the issue of which of the viruses caused the clinical symptoms of AGE has almost always been a challenge. Makimaa and colleagues (2020) reported that it is difficult to understand the possible interactions between different viruses, which can influence the clinical symptoms of AGE in multiple-virus infections [22]. Virus–virus interactions in human intestine are still poorly understood. Multiple-virus infections may be detrimental, insignificant, or beneficial for disease outcomes depending on the levels of interactions [23]. When multiple pathogens co-infect a host, they may be synergistically pathogenic, or one pathogen may influence the replication and disease severity caused by the other [24]. Apart from the direct interactions between co-infecting pathogens, host response also plays a pivotal role in shaping disease outcomes [25]. Future studies are essential to explore the effects of multiple-virus infections on the clinical symptoms of AGE in symptomatic patients. In our study, no differences were observed when comparing disease severity in this analysis. Taken together, our data not only increase awareness of the prevalence of multiple-virus infections in AGE, but they also contribute towards the effort to enhance our knowledge of clinical manifestations and disease severity between co-infecting viruses.

## 4. Materials and Methods

Fecal specimens were collected from all children with diarrhea who visited Hikita Pediatric Clinic in Kiryu city, Gunma prefecture, Japan, at any time from June 2014 to July 2017. Diarrhea was defined as ≥3 loose or watery feces per 24 h. The study population included 1245 children (52% male) with a median age of 2.6 years. At the time of clinic visits, these children did not have other disease conditions and no medications were being taken. Fecal specimens were stored at −30 °C until use. The viral genome was extracted by using a QIAamp Viral RNA mini kit (Qiagen, Hilden, Germany). Reverse transcription (RT) was performed with superscript reverse transcriptase III (Invitrogen, Carlsbad, CA, USA) with a random primer (Takara, Shiga, Japan). The specimens were analyzed using multiplex RT-PCR assays with three different primer sets. The first primer set could detect RVA, RVB, RVC, and HAdV, the second set NoV GI, NoV GII, HAstV, and SaV, and the third set AiV, PeV-A, and EV [26]. The data of the viruses detected in the study were entered into Microsoft Excel 2016 and were analyzed to evaluate the prevalence and temporal distribution of the viruses detected in the study. In addition, symptom data were collected using a standardized questionnaire and compared between single- and multiple-virus infections. Disease severity was assessed using a clinical Vesikari score. Pearson’s chi-squared (Χ^2^) test was used for the comparison of single-vs. multiple-virus infections with major symptoms such as fever (>38 °C), abdominal pain, vomiting, or diarrhea. The Mann–Whitney U test was used for a comparison of single- vs. multiple-virus infections with other clinical features such as the maximal degree and duration of fever or frequency per day and the duration of vomiting or diarrhea. Data were analyzed using the JMP statistical software program (SAS Institute; Cary, NC, USA). Differences with *p* < 0.05 were considered statistically significant. This study was performed with the approval of the Ethical Committee of Nihon University School of Medicine (No. 29-9-2) in accordance with the guidelines of the Declaration of Helsinki (World Medical Association). Informed consent was obtained from the parents of the children enrolled in this study.

## Figures and Tables

**Table 1:** Numbers and percentages of single- and multiple-virus infections in fecal specimens collected from children with diarrhea who visited Hikita Pediatric Clinic in Japan from June 2014 to July 2017.

Virus *	Total	Single Infection	(%)	Multiple Infection	(%)
NoV GII	1229	740	(60.2)	489	(39.8)
RVA	514	201	(39.1)	313	(60.9)
EV	241	99	(41.1)	142	(58.9)
PeV-A	152	62	(40.8)	90	(59.2)
HAdV	124	34	(27.4)	90	(72.6)
SaV	123	45	(36.6)	78	(63.4)
HAstV	122	39	(32.0)	83	(68.0)
NoV GI	22	5	(22.7)	17	(77.3)
AiV	2	0	(0)	2	(100)
RVC	1	0	(0)	1	(100)

* norovirus genogroup II (NoV GII), rotavirus A (RVA), enterovirus (EV), parechovirus A (PeV-A), human adeno-virus (HAdV), sapovirus (SaV), human astrovirus (HAstV), norovirus genogroup I (NoV GI), Aichi virus (AiV), and rotavirus C (RVC).

**Table 2:** Pairwise comparisons of the major symptoms were performed for the common single-virus infections and multiple-virus infections.

Infection	Fever	Abdominal Pain	Vomiting	Diarrhea
RVA/NoV GII vs. RVA	0.56	0.15	0.47	0.5
RVA/NoV GII vs. NoV GII	0.07	0.82	0.49	0.018 *
RVA vs. NoV GII	0.008 *	0.036 *	0.1	0.001 *
EV/NoV GII vs. EV	0.018 *	0.62	0.75	0.73
EV/NoV GII vs. NoV GII	0.13	0.43	0.0026 *	0.9
EV vs. NoV GII	<0.0001 *	0.12	0.0038 *	0.73
PeV-A/NoV GII vs. PeV-A	0.99	0.23	0.0003 *	0.72
PeV-A/NoV GII vs. NoV GII	0.32	0.032 *	0.88	0.32
PeV-A vs. NoV GII	0.19	<0.0001 *	<0.0001 *	0.0001 *
HAdV/NoV GII vs. HAdV	0.64	0.047 *	0.25	0.0088 *
HAdV/NoV GII vs. NoV GII	0.35	0.32	0.28	0.65
HAdV vs. NoV GII	0.83	0.092	0.015 *	0.0009 *

* Significant difference using Pearson’s chi-squared test.

**Table 3:** Pairwise comparisons of the major symptoms’ characteristics were performed for the common single-virus infections and multiple-virus infections.

Infection	Fever		Vomiting		Diarrhea		Vesikari
	Maximum	Duration	Frequency	Duration	Frequency	Duration	Score
RVA/NoV GII vs. RVA	0.35	0.68	0.51	0.71	0.57	0.65	0.91
RVA/NoV GII vs. NoV GII	0.11	0.24	0.28	0.61	0.0004 *	0.017 *	0.09
RVA vs. NoV GII	0.003 *	0.081	0.057	0.31	0.0013 *	0.002 *	0.1
EV/NoV GII vs. EV	0.25	0.3	0.81	0.93	0.38	0.43	0.52
EV/NoV GII vs. NoV GII	0.016 *	0.15	0.0094 *	0.014 *	0.6	0.94	0.66
EV vs. NoV GII	<0.0001 *	0.0024 *	0.0006 *	0.0023 *	0.52	0.24	0.68
PeV-A/NoV GII vs. PeV-A	0.67	0.96	0.0002 *	0.0034 *	0.0066 *	0.14	0.19
PeV-A/NoV GII vs. NoV GII	0.5	0.16	0.72	0.75	0.38	0.064	0.18
PeV-A vs. NoV GII	0.14	0.089	<0.0001 *	<0.0001 *	<0.0001 *	<0.0001 *	0.96
HAdV/NoV GII vs. HAdV	0.99	0.57	0.2	0.17	0.22	0.034 *	0.86
HAdV/NoV GII vs. NoV GII	0.13	0.6	0.089	0.23	0.82	0.32	0.57
HAdV vs. NoV GII	0.15	0.25	0.0031 *	0.0071 *	0.081	0.0003 *	0.78

* Significant difference using Mann–Whitney U test.

## Data Availability

Not applicable.

## References

[1] GBD 2017 Diarrhoeal Disease Collaborators (2020). Quantifying risks and interventions that have affected the burden of diarrhoea among children younger than 5 years: An analysis of the Global Burden of Disease Study 2017. Lancet Infect. Dis..

[2] Graves N.S. (2013). Acute gastroenteritis. Prim. Care.

[3] Wilhelmi I., Roman E., Sánchez-Fauquier A. (2003). Viruses causing gastroenteritis. Clin. Microbiol. Infect..

[4] Oppong T.B., Yang H., Amponsem-Boateng C., Kyere E.K.D., Abdulai T., Duan G., Opolot G. (2020). Enteric pathogens associated with gastroenteritis among children under 5 years in sub-Saharan Africa: A systematic review and meta-analysis. Epidemiol. Infect..

[5] Meier J.L. (2021). Viral acute gastroenteritis in special populations. Gastroenterol. Clin. N. Am..

[6] Bányai K., Estes M.K., Martella V., Parashar U.D. (2018). Viral gastroenteritis. Lancet.

[7] Omatola C.A., Olaniran A.O. (2022). Rotaviruses: From pathogenesis to disease control—A critical review. Viruses.

[8] Sridhar A., Karelehto E., Brouwer L., Pajkrt D., Wolthers K.C. (2019). Parechovirus A pathogenesis and the enigma of genotype A-3. Viruses.

[9] Chuchaona W., Khamrin P., Yodmeeklin A., Kumthip K., Saikruang W., Thongprachum A., Okitsu S., Ushijima H., Maneekarn N. (2017). Detection and characterization of Aichi virus 1 in pediatric patients with diarrhea in Thailand. J. Med. Virol..

[10] Yip C.C., Lo K.L., Que T.L., Lee R.A., Chan K.H., Yuen K.Y., Woo P.C., Lau S.K. (2014). Epidemiology of human parechovirus, Aichi virus and salivirus in fecal samples from hospitalized children with gastroenteritis in Hong Kong. Virol. J..

[11] Zhang S.X., Zhou Y.M., Xu W., Tian L.G., Chen J.X., Chen S.H., Dang Z.S., Gu W.P., Yin J.W., Serrano E. (2016). Impact of co-infections with enteric pathogens on children suffering from acute diarrhea in southwest China. Infect. Dis. Poverty.

[12] De Grazia S., Bonura F., Bonura C., Mangiaracina L., Filizzolo C., Martella V., Giammanco G.M. (2020). Assessing the burden of viral co-infections in acute gastroenteritis in children: An eleven-year-long investigation. J. Clin. Virol..

[13] Biscaro V., Piccinelli G., Gargiulo F., Ianiro G., Caruso A., Caccuri F., De Francesco M.A. (2018). Detection and molecular characterization of enteric viruses in children with acute gastroenteritis in Northern Italy. Infect. Genet. Evol..

[14] Arowolo K.O., Ayolabi C.I., Lapinski B., Santos J.S., Raboni S.M. (2019). Epidemiology of enteric viruses in children with gastroenteritis in Ogun State, Nigeria. J. Med. Virol..

[15] Nguyen T.A., Yagyu F., Okame M., Phan T.G., Trinh Q.D., Yan H., Hoang K.T., Cao A.T., Le H.P., Okitsu S. (2007). Diversity of viruses associated with acute gastroenteritis in children hospitalized with diarrhea in Ho Chi Minh City, Vietnam. J. Med. Virol..

[16] Chen Y., Li Z., Han D., Cui D., Chen X., Zheng S., Yu F., Liu J., Lai S., Yan Y. (2013). Viral agents associated with acute diarrhea among outpatient children in southeastern China. Pediatr. Infect. Dis. J..

[17] Akdag A.I., Gupta S., Khan N., Upadhayay A., Ray P. (2020). Epidemiology and clinical features of rotavirus, adenovirus, and astrovirus infections and coinfections in children with acute gastroenteritis prior to rotavirus vaccine introduction in Meerut, North India. J. Med. Virol..

[18] Japhet M.O., Famurewa O., Adesina O.A., Opaleye O.O., Wang B., Höhne M., Bock C.T., Mas Marques A., Niendorf S. (2019). Viral gastroenteritis among children of 0-5 years in Nigeria: Characterization of the first Nigerian aichivirus, recombinant noroviruses and detection of a zoonotic astrovirus. J. Clin. Virol..

[19] Lu L., Jia R., Zhong H., Xu M., Su L., Cao L., Dong Z., Dong N., Xu J. (2015). Molecular characterization and multiple infections of rotavirus, norovirus, sapovirus, astrovirus and adenovirus in outpatients with sporadic gastroenteritis in Shanghai, China, 2010–2011. Arch. Virol..

[20] Kim G.R., Kim S.H., Jeon G.W., Shin J.H. (2020). Prevalence of eleven infectious viruses causing diarrhea in Korea. Jpn. J. Infect. Dis..

[21] Ouédraogo N., Kaplon J., Bonkoungou I.J., Traoré A.S., Pothier P., Barro N., Ambert-Balay K. (2016). Prevalence and genetic diversity of enteric viruses in children with diarrhea in Ouagadougou, Burkina Faso. PLoS ONE.

[22] Makimaa H., Ingle H., Baldridge M.T. (2020). Enteric viral co-infections: Pathogenesis and perspective. Viruses.

[23] McArdle A.J., Turkova A., Cunnington A.J. (2018). When do co-infections matter?. Curr. Opin. Infect. Dis..

[24] Kumar N., Sharma S., Barua S., Tripathi B.N., Rouse B.T. (2018). Virological and immunological outcomes of coinfections. Clin. Microbiol. Rev..

[25] Devi P., Khan A., Chattopadhyay P., Mehta P., Sahni S., Sharma S., Pandey R. (2021). Co-infections as modulators of disease outcome: Minor players or major players?. Front. Microbiol..

[26] Thongprachum A., Takanashi S., Kalesaran A.F., Okitsu S., Mizuguchi M., Hayakawa S., Ushijima H. (2015). Four-year study of viruses that cause diarrhea in Japanese pediatric outpatients. J. Med. Virol..

